# A cluster‐randomized controlled trial to improve the quality of integrated HIV‐tuberculosis services in primary healthcareclinics in South Africa

**DOI:** 10.1002/jia2.25803

**Published:** 2021-09-08

**Authors:** Santhanalakshmi Gengiah, Pierre M. Barker, Nonhlanhla Yende‐Zuma, Mduduzi Mbatha, Shane Naidoo, Myra Taylor, Marian Loveday, Mesuli Mhlongo, Clark Jackson, Andrew J. Nunn, Nesri Padayatchi, Salim S. Abdool Karim, Kogieleum Naidoo

**Affiliations:** ^1^ Centre for the AIDS Programme of Research in South Africa (CAPRISA) Durban South Africa; ^2^ Institute for Healthcare Improvement Cambridge Massachusetts USA; ^3^ Department of Maternal and Child Health Gillings School of Global Public Health University of North Carolina Chapel Hill North Carolina USA; ^4^ MRC‐CAPRISA HIV‐TB Pathogenesis and Treatment Research Unit Doris Duke Medical Research Institute University of KwaZulu‐Natal Durban South Africa; ^5^ Department of Health Pretoria South Africa; ^6^ School of Nursing and Public Health University of KwaZulu‐Natal Durban South Africa; ^7^ HIV Prevention Research Unit South African Medical Research Council Durban South Africa; ^8^ Medical Research Council Clinical Trials Unit at University College London (UCL) London UK; ^9^ Department of Epidemiology Mailman School of Public Health Columbia University New York USA

**Keywords:** cluster‐randomized, collaboratives, HIV‐TB services, integration, primary healthcare clinics, quality improvement

## Abstract

**Introduction:**

: Tuberculosis (TB) remains the most common cause of death among people living with HIV. Integrating HIV and TB services reduces mortality but is sub‐optimally implemented. Quality improvement (QI) methods offer a low‐cost and easily implementable approach to strengthening healthcare delivery systems. This trial assessed a QI intervention on key process indicators for delivering integrated HIV‐TB care in rural South African primary healthcare (PHC) clinics.

**Methods:**

Sixteen nurse supervisors, (each with a cluster of clinics) overseeing 40 PHC clinics, were randomized 1:1 to the intervention or the standard of care (SOC) groups. The QI intervention comprised three key components: clinical and QI skills training, on‐site mentorship of nurse supervisors and clinic staff, and data quality improvement activities to enhance accuracy and completeness of routine clinic data. The SOC comprised monthly supervision and data feedback meetings. From 01 December 2016 to 31 December 2018, data were collected monthly by a team of study‐appointed data capturers from all study clinics. This study's outcomes were HIV testing services (HTS), TB screening, antiretroviral therapy (ART) initiation, isoniazid preventive therapy (IPT) initiation and viral load (VL) testing.

**Results:**

The QI group (eight clusters) comprised 244 clinic staff who attended to 13,347 patients during the trial compared to the SOC group (eight clusters) with 217 clinic staff who attended to 8141 patients. QI mentors completed 85% (510/600) of expected QI mentorship visits to QI clinics. HTS was 19% higher [94.5% vs. 79.6%; relative risk (RR)=1.19; 95% CI: 1.02–1.38; *p*=0.029] and IPT initiation was 66% higher (61.2 vs. 36.8; RR=1.66; 95% CI: 1.02–2.72; *p*=0·044), in the QI group compared to SOC group. The percentage of patients screened for TB (83.4% vs. 79.3%; RR=1.05; *p*=0.448), initiated on ART (91.7 vs. 95.5; RR=0.96; *p*=0.172) and VL testing (72.2% vs. 72.8%; RR=0.99; *p*=0.879) was similar in both groups.

**Conclusions:**

QI improved HIV testing and IPT initiation compared to SOC. TB screening, ART initiation and VL testing remained similar. Incorporating QI methods into routine supervision and support activities may strengthen integrated HIV‐TB service delivery and increase the success of future QI scale‐up activities.

## 1 INTRODUCTION

In South Africa (SA), the convergence of the HIV and tuberculosis (TB) epidemics created one of the largest HIV‐TB co‐epidemics in the world [1]. In 2016, an estimated 59% of newly diagnosed TB patients were co‐infected with HIV and the TB mortality rate in HIV‐TB co‐infected patients was 180 per 100,000 people, compared to 41 per 100,000 in HIV‐negative TB patients people [1]. To reduce TB‐related mortality in people living with HIV, the World Health Organization recommended integration of TB and HIV treatment and care services, hereafter written as HIV‐TB services [2]. In practice, this translates to making both HIV and TB services available to patients at the same facility, on the same visit day, by the same clinic team [2]. In resource‐constrained settings, HIV‐TB services optimally utilize very limited healthcare resources, are known to improve AIDS‐free survival and preferred by patients as a cost‐ and time‐saving strategy [2, 3, 4].

**Box 1: Package of integrated HIV‐TB services**

■ Testing and counselling for HIV in all patients with TB
■ Intensified case finding for TB in HIV‐infected patients
■ IPT for HIV‐positive patients who screen TB negative
■ ART initiation for all HIV‐TB co‐infected patients
■ CPT for HIV‐TB co‐infected patients
■ Enhanced retention in care strategies
■ Enhanced ART and TB treatment adherence strategies, including, viral load testing coverage
■ A fully integrated data management system — adopting the approach of one patient, one appointment, one file and one data management system

ART, antiretroviral therapy; CPT, cotrimoxazole preventive therapy; IPT, isoniazid preventive therapy; TB, tuberculosis; VL, viral load

By 2016, HIV‐TB services were routine care in SA and comprised: early antiretroviral therapy (ART) for TB patients irrespective of CD4 cell count; isoniazid preventive therapy (IPT) for eligible HIV patients; HIV testing services (HTS) for all patients, especially TB patients; TB screening and diagnostic testing [5]. Evidence has surfaced of patients accessing primary healthcare (PHC) clinics and being missed for HIV and TB services [6, 7, 8, 9]. Integrated HIV‐TB service delivery requires high‐level organization and planning by clinic teams against a backdrop of large patient numbers and constrained resources [6, 7, 10, 11]. Innovative solutions to strengthen systems for HIV‐TB service delivery are needed [12].

Effective strategies to improve integrated HIV‐TB service delivery are unknown [13]. Quality improvement (QI) offers a potential approach for consideration due to its focus on improving underlying systems and engaging PHC staff to identify practical, low‐cost solutions to address deficiencies with available resources. [14, 15] QI interventions to reduce mother‐to‐child HIV transmission and mortality have been successful in many African countries [16, 17]. Little is known of the effectiveness of QI to impact HIV‐TB services [12].

Evaluations of QI effectiveness have rarely been conducted within a randomized controlled trial. Given the considerable commitment of time, effort, financial and human resources dedicated to implementing QI, rigorous testing of the approach is warranted. The Centre for the AIDS Programme of Research in South Africa (CAPRISA) conducted the *Scaling up TB HIV integration* (SUTHI) trial, which tested the effectiveness of a QI intervention in improving HIV‐TB services to reduce mortality in HIV and HIV‐TB patients. This paper assesses the effectiveness of QI to improve process indicators of HIV‐TB service delivery compared to standard support and supervision.

## 2 METHODS

### 2.1 Study design

This is a nested sub‐study within the SUTHI trial. The SUTHI trial design was published elsewhere [12]. SUTHI was a cluster‐randomized trial that tested the effectiveness of a QI intervention to improve HIV‐TB service delivery in reducing TB‐related mortality among HIV, TB and HIV‐TB patients. The trial was conducted between 01 December 2016 and 31 December 2018. At the PHC level in SA, nurse supervisors typically oversee 3–5 PHC clinics. In the SUTHI trial, the ‘clusters’ were the nurse supervisors. PHC clinics were assigned to the same study arm as their respective nurse supervisor and followed up for 18 months. The primary outcome of the SUTHI trial was all‐cause mortality among HIV, TB and HIV‐TB patients. This nested sub‐study evaluated a set of process indicators that typically comprise integrated HIV‐TB service delivery.

### 2.2 Study setting

**Figure 1:**
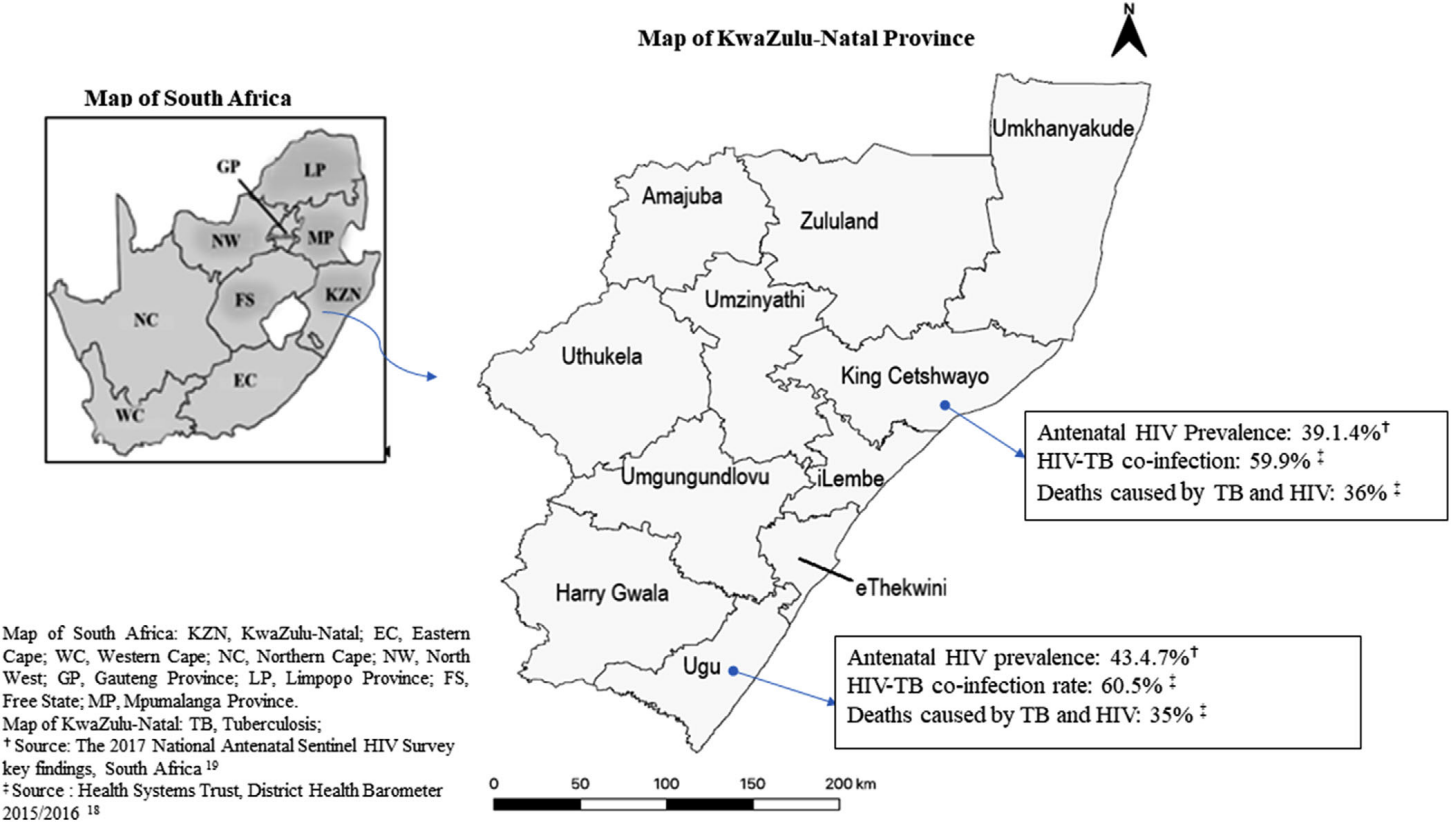
Map of KwaZulu‐Natal Province in South Africa.

The SUTHI trial was conducted in two predominantly rural districts, the Ugu and King Cetshwayo District (KCD), in KwaZulu‐Natal (KZN) Province, SA. Figure 1 shows the study districts and summarizes the burden of HIV and TB. HIV and TB are responsible for over a third of all deaths in Ugu and KCD, 35% and 36%, respectively [18, 19].

### 2.3 Randomization

**Figure 2:**
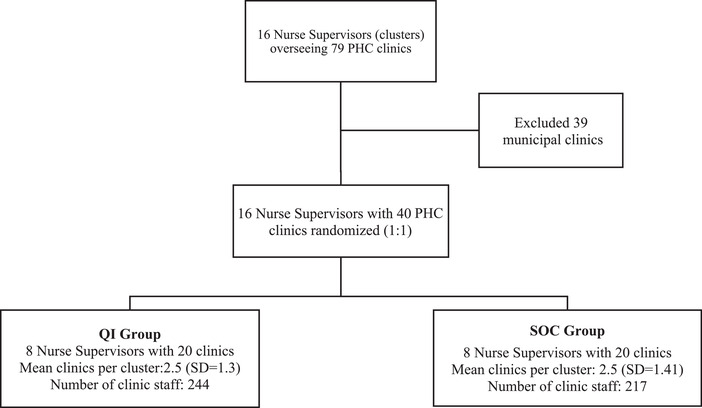
Randomization of nurse supervisors and respective clinics.

The KZN District Health Offices provided a list with a total of 16 nurse supervisors for the Ugu district and KCD. Study eligibility criteria of nurse supervisors and clinics have been published elsewhere [12]. The main criterion was acquiring verbal agreement of nurse supervisors and nurses‐in‐charge of individual clinics for study participation. The study statistician randomized supervisors in a 1:1 ratio using computer‐generated randomization. Clinics classified as municipal clinics were automatically excluded as their management and resource allocation were different to those of typical PHC clinics (Figure 2). No nurse supervisors or clinics declined or withdrew their participation.

### 2.4 Study intervention

**Figure 3:**
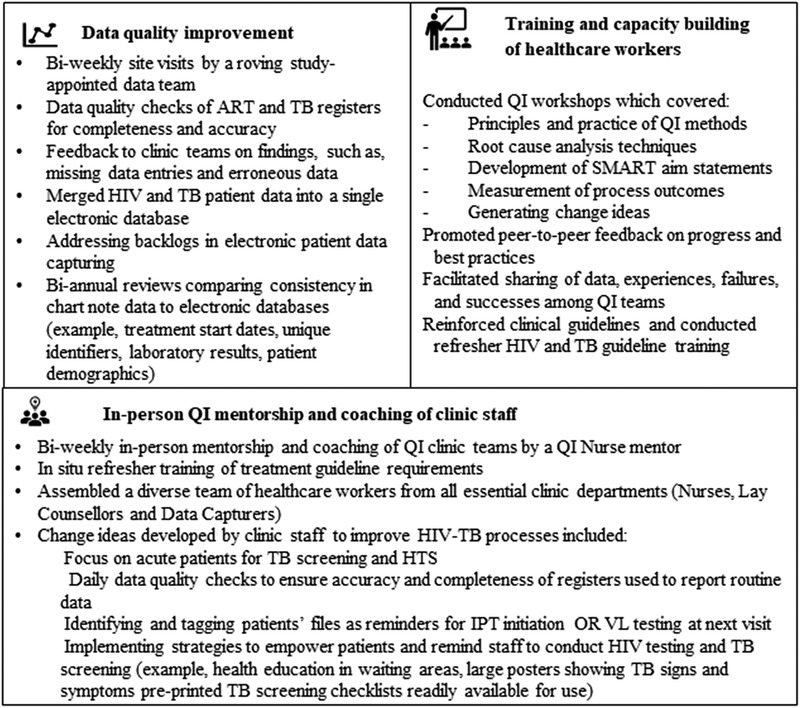
The three‐component quality improvement intervention.

The QI intervention comprised three essential components delivered as a ‘package’: (1) training and capacity building of healthcare workers; (2) in‐person QI mentorship of clinic staff; and (3) data quality improvement (DQI) activities to enhance reliability of routine clinic data. Figure 3 provides detail on each component.

The QI intervention was structured as a Breakthrough Series Collaborative [20]. Nurse supervisors and their respective clinics formed a ‘collaborative’. The collaborative met at three QI workshops, timed at 6‐month intervals, for QI and clinical skills training, and shared experiences and best practices [20]. At least one member of each department within a clinic (i.e. nurses, lay counsellors and data capturers) and nurse supervisors participated in QI workshops which were interactive and promoted group work.

**Figure 4:**
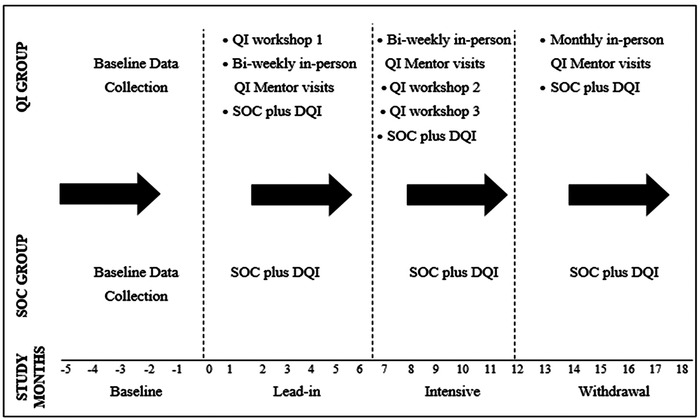
Study procedures and sequence of events.

Between QI workshops, a study‐appointed QI nurse mentor conducted in‐person mentorship visits to clinics to reinforce workshop content, review clinic data and guide change idea development. Figure 4 illustrates the timing of workshops and mentorship visits. The Plan‐Do‐Study‐Act cycle was the guiding framework to develop, test and improve upon change ideas for HIV‐TB service delivery. Box 1 lists the set of routine HIV‐TB integration services that the collaborative aimed to improve [12].

Lastly, DQI activities were conducted to ensure that the most accurate and complete data possible were available to QI clinic teams to drive the QI process and for research purposes (Figure 3).

### 2.5 Standard support and supervision

All study clinics received standard support for all health services, including HIV and TB services. Standard support activities comprised: (1) monthly clinic‐based visits by the nurse supervisor; (2) quarterly visits by the District Management Team (DMT), usually represented by the TB and HIV/ART/STI/TB (HAST) Managers from the district health office; and (3) monthly performance monitoring and feedback meetings hosted by the DMT to identify gaps in HIV and TB service delivery. Supervisory visits typically consisted of file and summary data reviews, with feedback to senior clinic management. In April 2016 (8 months prior to the SUTH trial), the Department of Health (DOH)‐initiated monthly performance monitoring meetings called ‘Nerve Centre Meetings’ in both districts. These mandatory meetings were the key mechanism through which facilities received feedback on performance and were typically attended by at least one representative from each facility. Assistance to clinics by local non‐governmental organizations (NGOs) is common in the South African healthcare context. Prior to and during the study, PHC clinics in both districts received technical support from local NGOs, such as direct patient care, clinical and data management training. DQI activities were conducted in standard of care (SOC) clinics to ensure comparability in data between both groups.

### 2.6 Study procedures and phases

Figure 4 illustrates the timing of study activities in both study groups. Baseline was defined as the period 6 months prior to study enrolment. The 18‐month follow‐up period was divided into three phases of 6‐months duration, and each phase contained a different level of QI support. The *lead‐in phase* was the period from months 1 to 6, when the first of three QI workshops was completed, and bi‐weekly QI mentor visits commenced. The *intensive phase* was the period from months 7 to 12 when the second and third QI workshops were completed, and bi‐weekly QI mentor visits continued. The QI intervention was at its maximum strength in this phase. The *withdrawal phase* was the period from months 13 to 18 with minimal QI support, reduced to once‐a‐month visits.

Two study‐appointed QI mentors were expected to each make at least 30 QI visits per clinic during the study. This comprised 24 QI visits (two visits per month) in the lead in and intensive phases and six QI visits (one visit per month) in the withdrawal phase.

From 01 December 2016 to 31 December 2018, data were collected monthly by study‐appointed data capturers. Paper‐based registers, patient charts and patient electronic databases were the data sources for HIV‐TB process indicators. Quarterly reports from the National Health Laboratory Service and Electronic TB Register were used to assess the number of sputum samples sent for TB diagnostic tests and confirmed TB patients. Summaries of data were recorded on a study data collection form and transmitted via fax to a central database.

### 2.7 Study outcomes

For this sub‐study, we assessed changes in key process indicators representative of integrated HIV‐TB healthcare service delivery. Table 1 lists and defines the process indicators and the data elements (numerators and denominators) that were used to calculate proportions of patients who were eligible for and received HIV‐TB services. HIV‐TB process indicator performance was aggregated at the month‐level. Patients who received a service (counted in the numerator) are a sub‐group of the patients who were eligible for the service (counted in the denominator). Occasionally, patients received the service in the next month and were subsequently added to the previous month's data.

**Table 1:** Definitions of HIV‐TB process indicators

HIV‐TB services	HIV‐TB process indicator	Data elements used to express process indicators as a proportion	Primary data sources^c^
HTS for PHC clinic attendees	Proportion of patients who accessed HIV tests, as a percentage of the clinics’ HIV testing target	Numerator: Number of patients tested for HIV	HTS Register
		Denominator: Clinic assigned target for HTS^a^	
	Proportion of new TB patients tested for HIV	Numerator: Number of new TB patients tested for HIV	ETR
		Denominator: Number of new TB patients	
	Proportion of new TB patients tested HIV positive	Numerator: Number of TB patients tested HIV positive	
		Denominator: Number of new TB patients tested for HIV	
TB screening among PHC clinic attendees (TB screening)	Proportion of clinic attendees screened for TB signs or symptoms	Numerator: Number of clinic attendees screened for TB signs and symptoms (adults and children)	TB screening register
		Denominator: Clinic headcount^b^	
Confirmed new TB cases	Proportion of Xpert MTB/RIF tests with a ‘TB detected’ outcome	Numerator: Xpert MTB/RIF tests with a ‘TB detected’ outcome	NHLS
		Denominator: Number of sputum samples collected for Xpert MTB/RIF testing for initial TB diagnosis	
TB confirmed patients initiated onto TB treatment	Proportion of patients with a TB confirmed Xpert MTB/RIF^#^ result initiated onto TB treatment	Numerator: Number of patients initiated onto TB treatment	ETR
		Denominator: Number of patients with a ‘TB detected’ MTB/RIF result	
IPT initiation among eligible new ART patients (IPT initiation)	Proportion of new ART patients initiated onto IPT	Numerator: Number of new ART patients initiated on IPT	Patient file
		Denominator: Number of new ART patients with no signs or symptoms of TB	
ART initiation among HIV‐TB co‐infected patients	Proportion of HIV‐TB services co‐infected patients initiated on ART	Numerator: Number of HIV‐TB co‐infected patients initiated on ART	ART register
		Denominator: Number of confirmed TB patients tested positive for HIV	
VL testing at month 12 after ART initiation (VL testing)	Proportion of eligible ART patients who had a VL test 12 months after initiating ART	Numerator: Number of ART patients who received a VL test at month 12 after ART initiation	TIER.Net
		Denominator: Number of ART patients eligible for a VL test at month 12 after ART initiation	

Abbreviations: ART, antiretroviral therapy; ETR, Electronic TB Register; HTS, HIV testing services; IPT, isoniazid preventive therapy; NHLS, National Health Laboratory Services; PHC, primary healthcare; TIER, Three Integrated Electronic Registers; TB, tuberculosis; VL, viral load.

Xpert MTB/Rif, a rapid, molecular, cartridge‐based test used for tuberculosis diagnostics that provides an immediate rifampicin resistance result.

^a^ All primary healthcare clinics are given an HIV testing services target each year by the respective District Health office. Targets were calculated based on HIV prevalence and patient population within a clinic's catchment area.

^b^ Number of people accessing any health services at a facility during a specified period.

^c^ Data sources listed were considered the primary source of data but if necessary other data sources were used to verify data.

## 3 STATISTICAL ANALYSIS

In this study, the cluster was the unit of analysis, hence, all clinics and its respective patients in a cluster we considered as one unit. Study group proportions per study phase were calculated as follows: First, the proportions per cluster were calculated by summation of numerators divided by the sum of the denominators of all respective clinics in a cluster per month. A proportion of zero was replaced with 0.00001 (or 0.001 when using percentages). If a denominator was zero (i.e. no one was eligible), then that month was ignored. Second, we calculated cluster‐specific geometric means (GM) across months associated with a phase (Figure 4). Third, study group‐specific GM were calculated as cluster‐specific proportions per phase. An unpaired *t*‐test was used to compare the study groups.

The relative risk (RR) between study groups was calculated to provide a measure of the improvement within each phase. Changes in HIV‐TB process indicator performance between baseline and intensive phase are shown as the QI intervention was at its maximum strength during this phase (Figure 4). In a post‐hoc analysis, HIV‐TB process indicators of interest were stratified by cluster‐specific patient volume to understand how results varied within clusters of different sizes. We sorted cluster‐specific patient volume into three categories with the following ranges: category 1 included cluster headcounts of less than or equal to 2500 (<2500), category 2 included cluster headcounts of greater than 2500 and less than or equal to 3500 (> 2500 <3500) and category 3 included cluster headcounts of greater than 3500 (>3500). Statistical analyses were performed using SAS (SAS Institute, Cary, NC, USA) version 9.4.

### 3.1 Ethics and gatekeeper permissions

The SUTHI trial was approved by the Biomedical Research Ethics Committee of the University of KwaZulu‐Natal (BF108/14). Participant informed consent was waivered for this study. The KZN Health Research and Knowledge Management committee granted permission to access PHC clinics in the study districts.

## 4 RESULTS

Between 01 April 2016 and 30 June 2016, 16 nurse supervisors and 79 clinics under their oversight were screened for the SUTHI trial. All nurse supervisors agreed to participate; however, 39 municipal clinics were ineligible, hence, 40 clinics were included in the randomization (Figure 2). Eight nurse supervisors overseeing 20 clinics were randomized to the QI group and 16 nurse supervisors overseeing 20 clinics were randomized to the SOC group. In the QI group, 244 clinic staff who served 13,347 HIV and HIV‐TB patients were exposed to QI mentorship. In the SOC arm, 217 PHC staff, who served 8141 HIV and HIV‐TB patients, received standard support and supervision. The mean headcount was 3448.8 [Standard Deviation (SD)=1833.1%] and 70% (14/20) of clinics were high‐burden in the QI group compared to a mean headcount of 2836.4 (SD=993.8) and 55% (11/20) high‐burden clinics in the SOC group (Table 2). Table 3 shows the proportion of completed visits per QI group cluster. QI mentors completed 85% (510/600) of expected visits. Completed visits across the eight clusters ranged from 77% to 100%.

**Table 2:** Baseline characteristics of the quality improvement (QI) group and standard of care (SOC) group clusters

	QI group	SOC group
Patients in care, mean per month (SD)		
Patient headcount^a^	3448.8 (1833.1)	2836.4 (993.8)
HIV patients in care	1047.6 (1250.45)	653·0 (443.3)
HIV‐TB patients in care	133.8 (128.5)	84.7 (60.3)
Clinic categorization *n*/*N* (%)^b^		
High‐burden clinics	14/20 (70%)	11/20 (55%)
Low‐burden clinics	6/20 (30%)	9/20 (45%)
Staff complement (*n*)		
NIMART trained nurses^c^	79	79
TB trained nurses^d^	29	39
Enrolled nurses	27	17
Data capturers	30	29
Lay counsellors	43	38
Community caregivers	274	286
Nurse:patient ratio		
Monthly nurse:patient ratio	1:308	1:266

Abbreviations: NIMART, Nurse Initiated Management of Antiretroviral Therapy; QI, quality improvement; SD, Standard Deviation; SOC, standard of care; TB, tuberculosis.

^a^ Refers to all patients accessing the clinic for any care service.

^b^ Mean monthly patient headcount >2500 = High burden, < 2500 =Low burden.

^c^ Refers to nurses who are initiating and managing patients on ART after undergoing the necessary training provided by an appropriate service provider. NIMART training was not provided in the study.

^d^ Refers to nurses who are initiating and managing TB patients after undergoing the necessary training provided by an appropriate service provider. Training for TB treatment initiation and management of TB patients was not provided in the study.

**Table 3:** Expected quality improvement (QI) visits completed in the QI group clusters

QI group clusters				
Cluster	Number of clinics (*n*)	Actual visits per cluster (*n*)	Expected visits per cluster (*N*)	Percentage of expected visits completed (%)
I1	1	25	30	83
I2	1	26	30	87
I3	3	73	90	81
I6	3	84	90	93
I7	4	92	120	77
I8	1	30	30	100
I12	4	100	120	83
I14	3	80	90	89
Total	20	510	600	85

Abbreviations: I, intervention (i.e. the QI group); QI, quality improvement.

The QI intervention addressed five of the eight HIV‐TB services in Box 1, specifically: HTS, TB screening, IPT initiation, ART initiation in HIV‐TB co‐infected patients and viral load (VL) testing at 12 months on ART. An integrated electronic TB and HIV data systems was rolled‐out at the start of the trial and implemented in all clinics. Missing data and limited study resources were barrier to addressing cotrimoxazole preventive therapy (CPT) and retention in care.

Table 4 compares the performance of the QI and SOC groups at the baseline and intensive phases.

**Table 4:** Comparison of HIV‐TB service delivery between quality improvement and standard of care groups

	QI group		SOC group		RR (95% CI)	*p*‐value
	*N*	Percentage (95% CI)	*N*	Percentage (95% CI)		
HTS for PHC clinic attendees						
Baseline	40,184	84.8 (75.5–95.3)	28,666	85.3 (74.9–97.2)		
Intensive phase	35,164	94.5 (91.9–97.1)	32,839	79.6 (68.7–92.3)	1.19 (1.02–1.38)	0.029*
HTS in TB patients						
Baseline	984	88.7 (79.6–98.9)	581	85.7 (78.3–93.7)		
Intensive phase	917	92.8 (88.3–97.4)	542	91.3 (87.1–95.7)	1.02 (0.96–1.08)	0.589
TB screening for PHC clinic attendees						
Baseline	470,192	76.2 (65.4–88.9)	360,028	78.9 (68.3–91.1)		
Intensive phase	442,127	83.4 (76.5–90.9)	354,339	79.3 (70.1–89.8)	1.05 (0.92–1.21)	0.448
ART initiation among HIV‐TB patients						
Baseline	657	95.8 (93.3–98.3)	380	98.9 (97.6–100.0)		
Intensive phase	547	91.7 (86.3–97.4)	333	95.5 (93.1–98.0)	0.96 (0.90–1.02)	0.172
Initiating isoniazid preventive therapy (IPT) among eligible new ART patients						
Baseline	5004	15.9 (4.8–52.5)	2739	27.7 (16.2–47.1)		
Intensive phase	3138	61.2 (50.6–74.1)	1884	36.8 (22.8–59.4)	1.66 (1.02–2.72)	0.044*
VL testing at month 12 after ART initiation						
Baseline	3082	61.4 (56.4–66.8)	2183	57.5 (45.7–72.4)		
Intensive phase	4663	72.2 (65.0–80.1)	2816	72.8 (66.4–79.8)	0.99 (0.87–1.12)	0.879
Additional outcomes						
Confirmed new TB cases, % (*n*)						
Baseline	6720	8.7 (583)	4655	7.9 (369)	0.8	^*^
Intensive phase	6007	9.9 (598)	4531	8.1 (365)	1.8	^*^
TB confirmed patients initiated onto TB treatment, % (*n*)						
Baseline	583	98.5 (574)	369	93.8 (346)	4.7	^*^
Intensive phase	598	87.5 (523)	365	88.5 (323)	–1.0	^*^

Abbreviations: ART, antiretroviral therapy; CI, confidence interval; HTS, HIV testing services; IPT, isoniazid preventive therapy; PHC, primary healthcare; QI, quality improvement; RR, relative risk; SOC, standard of care; TB, tuberculosis; VL, viral load.

* *p*‐value significant at <0.05.

^†^ Only quarterly summary data were available.

**Figure 5:**
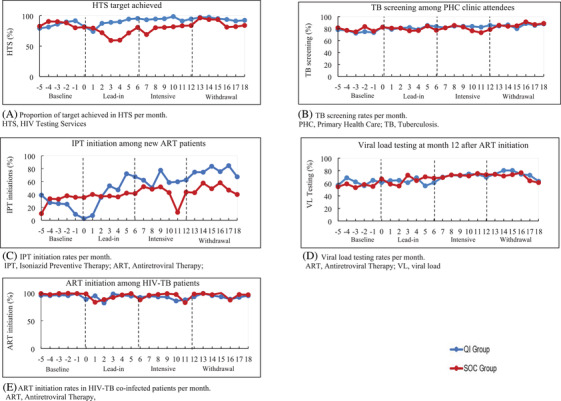
HIV‐TB process indicator performance in quality improvement and standard of care groups.

At baseline, both groups were similar in performance for all process indicators. The QI group improved HTS by 9.7% from 84.8% (95% CI: 75.5–95.3) to 94.5% (95% CI: 91.9–97.1), compared to a decline of 5.7% from 85.3% (95% CI: 74.9–97.2) to 79.6% (95% CI: 68.7–92.3) in the SOC group. By the intensive period, HTS was 19% higher in the QI group than in the SOC group (94.5% vs. 79.6%; RR=1.19; 95% CI: 1.02–1.38; *p*=0.029). Figure 5a concurs with this finding and shows higher monthly HTS performance in the QI group between months 0 and 13. Thereafter, the QI group maintained its performance and the SOC group increased its performance (Figure 5a).

At baseline, IPT initiation rates in the QI and SOC groups were 15.9% (95% CI: 4.8–52.5) and 27.7% (95% CI: 16.2–47.1), respectively. By the intensive phase, IPT initiation rates were 61.2% (95% CI: 50.6–74.1) and 36.8% (95% CI: 22.8–59.4) in the QI and SOC groups, respectively, RR=1.66; 95% CI: 1.02–2.72; *p*=0.044 (Table 4). Table S1 shows the study groups’ performance in the lead‐in and withdrawal phases. In the withdrawal phase, the QI group achieved IPT initiation rates of 76.4% (95% CI: 66.3–88.1), compared to 50.8% (95% CI: 36.2–71.2) in the SOC group, RR=1.51; 95% CI: 1.06–2.14; *p*=0.026. Figure 5c illustrates the sustained higher improvement in the QI group during the study.

TB screening, ART initiation in HIV‐TB patients and VL testing in QI compared to SOC groups were (83.4% vs. 79.3%; RR=1.05; 95% CI: 0.92–1.21; *p*=0.448), (91.7 vs. 95.5; RR=0.96; 95% CI: 0.90–1.02; *p*=0.172) and (72.2 vs. 72.8; RR=0.99; 95% CI: 0.87–1.12; *p*=0.879), respectively (Table 4). Figures 5b–e illustrate the similarity in monthly performance between the study groups.

### 4.1 Post‐hoc analysis

Figure S1 shows the IPT initiation rates for QI and SOC clusters sorted into three categories representing patient volume. Of the eight QI clusters, four were classified as category 1 and four as category 3. Of the eight clusters in the SOC group, one was classified as category 1, six as category 2 and one as category 3.

In the QI group, category 1 clusters had baseline IPT initiation rates that ranged from 0.9% to 22.7% and the size of improvement ranged from 30.4% to 68.3% (Figure S1). Category 3 clusters had baseline IPT initiation rates that ranged from 35.8% to 45.0% and size of improvements ranged from 3.4% to 54.7%. In the SOC group, the category 1 cluster had a baseline IPT initiation rate of 8.7% and improved to 10.0%. Category 2 clusters and the one category 3 cluster made improvements in IPT initiations that ranged from 10.6% to 21.7% and 29.7%, respectively.

Figure S2 shows cluster‐specific HTS rates for QI and SOC clusters. In the QI group, category 1 clusters achieved baseline HTS rates which ranged from 85.2% to 98.6% and improvement rates that ranged from 0.8% to 29.7%; category 3 clusters achieved baseline rates of 64.7–90.7% and improvement sizes ranged from 0.8% to 29.7%. In the SOC group, categories 1, 2 and 3 were 83.6%, 63.2–100% and 79.4%, respectively, at baseline. In category 2, five clusters showed decreases in HTS rates, which ranged from 0.5% to 20.8%.

## 5 DISCUSSION

This trial demonstrated the effectiveness of QI interventions in improving two key HIV‐TB services, HTS and IPT initiation. The QI intervention did not significantly improve ART initiation in TB patients, TB screening and VL monitoring compared to the SOC group. CPT and retention in care for HIV‐TB patients were not addressed by the intervention because resources required to locate and capture large amounts of missing data were beyond the budget and time frame of the study. Instead, the study leadership took a decision to focus on indicators for which data were adequately available and improvement activities would make a meaningful impact.

The QI group's improvement of IPT initiations can be attributed to low baseline performance that offered large room for improvement and a comprehensive set of change ideas, which included: identification of a common time to start IPT after ART initiation (either 7, 14 or 30 days); development of an early identification system for patients eligible for IPT (e.g. tagged patient files); TB screening refresher training to boost nurses’ confidence to rule out TB; and clarifying staff responsibilities for IPT recording, stock control and data quality checks. In the QI group, small clusters made larger improvements in IPT initiation than large clusters, likely due to better coordination of efforts. HTS is a well‐established service within the public health sector and intervention generated an appreciable increase in HTS rates in larger clusters. Change interventions, such as group pretest counselling in waiting areas and targeting acute patients, maximized the larger clinics’ ability to offer HTS to large numbers of patients accessing the facility.

In SA, ART coverage among TB patients is 88%, an indication of the successful ART programme scale‐up and strong national policy. The pre‐existing high performance precluded our ability to show an impact of QI for this service [21]. For TB screening, proportions were reduced due to over‐inflated headcount data (the denominator) that erroneously included patients’ caregivers or accompanying family members not accessing services at the clinic. Despite DQI efforts, this data inaccuracy persisted in the study.

The QI intervention created a ‘demand’ for VL test completion reports, which are generated from electronic patient databases, and were only as accurate as the data entered. Backlogs in data capture prevented generation of timely and trustworthy reports. We dedicated approximately 6 months to addressing VL data backlogs which limited the time available to effectively address VL testing. Tracing patients to return for VL tests was resource‐intensive and required already scarce human and infrastructure resources.

Improvements in HIV‐TB service delivery after QI implementation have been observed in other studies. A Thai study evaluated QI in HIV care services between 2002 and 2008, and showed 75.0% improvement in TB screening (24.0– 99.0%) [22]. The size of improvement is likely due to introduction of new TB services rather than strengthening pre‐existing services as per our project. A Namibian QI program had similar TB screening improvement (81.0–87.0%) to our study, but attained modest IPT initiation improvements (16–28%) [23].

We acknowledge the impact of the DMTs in SOC group improvements. A Ugandan study showed performance feedback to be an effective intervention in improving TB services, however, was unable to establish its sustainability [24]. Our study demonstrated sustained improvement in SOC group clinics. The influence of the DMTs is observed in HTS, particularly a rapid improvement in HIV testing after a notable decline between months 1 and 6 (Figure 5a); however, TB screening and VL testing remained unchanged. IPT initiations improved and were sustained in the SOC group; however, the size of improvements was lower than in the QI group. While the DMTs were effective in making improvements, QI methods intensified that improvement.

We recommend that future scale‐up activities should initially target poor performing indicators to showcase the large improvements that are possible with QI and use these early successes to attract more clinics or districts to adopt QI. A systematic review of 27 QI collaboratives found that baseline performance levels in indicators <50% were 10 times more likely to reach levels of >80% [25]. Implementers of scale‐up should consider directing more resources and support to large clinics, particularly if interventions required are complex. Well‐established services should still be considered for improvement to encourage re‐assessment of ingrained systems that could benefit from revitalization. Lastly, the affordability and sustainability of QI interventions may be enhanced if DMTs (or similar group in other settings) complemented performance feedback with the structure, strategies and tools offered by QI.

QI collaboratives, as a scale‐up approach, have been widely adopted in high‐income countries and have rapidly spread to low‐ and middle‐income countries [26, 27]. However, costs associated with implementing collaboratives are a potential scale‐up barrier [28]. Cost considerations, specifically at the start‐up phase, include face‐to‐face meetings, in‐person mentorship visits, clinicians’ time spent on clinical skills training, baseline data clean‐up and analysis, coordination of QI collaborative activities, and administrative and personnel support [28, 29]. Encouragingly, studies show that QI collaboratives can be cost‐effective in improving implementation of clinical guidelines for acute and chronic conditions [28]. The benefit to large populations and reduced need for expensive treatment and high‐care are cost savings that outweigh the costs of the QI collaborative itself [28].

In SA, a scale‐up strategy for QI collaboratives to improve HIV‐TB services is achievable with optimal use of resources and systems, namely the Nerve‐Centre meetings. Successful scale‐up requires a national leader to manage and coordinate activities. To this end, local NGOs have an important role to play. A previous partnership between the SA DOH and a network of NGOs to improve prevention of mother‐to‐child HIV transmission was highly successful [30]. In Table S2, we outline the scale‐up activities and resource inputs needed, namely: (1) partnership between the SA DoH and NGOs, (2) development of a best‐practices package; (3) skilled QI trainers to build QI capacity; and (4) mechanisms for distribution and access to QI training and tools.

This study had limitations. Larger clusters were randomized to the QI group, which may have been prevented if Nurse Supervisors were matched by patient volume. Matching was not possible as groups of clinics were assigned to Nurse Supervisors by the SA DOH, driven largely by geographic location. Further, matching of clusters would have introduced limitations in conducting analyses (loss of degrees of freedom) and in making statistical inferences. Contamination between the QI and SOC group clinics cannot be ruled out. Highly motivated DMTs frequently and consistently reviewed data with study clinics and were privy to QI trainings and materials. Both QI and SOC group staff attend DMT meetings and sharing of ideas and best practices were unavoidable and potentially reduced the true difference between the groups.

## 6 CONCLUSIONS

QI interventions were effective in improving HTS and IPT initiations. Contexts where performance feedback is a routine practice likely enhance the success of QI interventions. QI methods can complement and strengthen standard supervision and support; however, poor data quality is a threat to the success of QI interventions.

## COMPETING INTERESTS

The authors declare they have no competing interests.

## AUTHOR CONTRIBUTIONS

SG led the implementation of the study, data validation and cleaning, wrote the original draft and interpreted results. KN acquired funding for the study and is the grant holder, had study oversight and contributed to writing and editing the manuscript. SSAK, PMB and AJN contributed to the study design, intervention design and edited the manuscript. NYZ, MM1 and CJ conducted data analysis verification, interpretation and reviewed and edited the manuscript. MM, SN, MT, ML and NP interpreted the results and reviewed and edited the manuscript for critical intellectual content. MM1 and SG validated the data and conducted analyses. All authors have read and approved the final manuscript.

## FUNDING

The research reported in this paper was supported by the South African Medical Research Council with funds received from the South African National Department of Health, and the UK Medical Research Council, with funds received from the UK governments Newton Fund.

This UK‐funded award is part of the EDCTP2 programme supported by the European Union.

## DISCLAIMER

The funder of the study had no role in the study design, data collection, data analysis, data interpretation or writing of the manuscript. The corresponding author had full access to all the data in the study and had final responsibility for the decision to submit for publication.

## Supporting information

Supporting Information
