# Supplementary material for: A cluster‐randomized controlled trial to improve the quality of integrated HIV‐tuberculosis services in primary healthcareclinics in South Africa
Source: J Int AIDS Soc. 2021 Sep 8;24(9):e25803. doi: 10.1002/jia2.25803 (PMC8426757; doi:10.1002/jia2.25803)
Supplement: Supplementary file 1 — Supporting Information [file JIA2-24-e25803-s001.docx]

|  | Percentage (95% CI) | | RR (95% CI) | p-value |
| --- | --- | --- | --- | --- |
|  | QI Group | SOC Group |  |  |
| HTS for PHC clinic attendees | | | | |
| Lead-in | 88.0  (81.3 – 95.3) | 70.0  (55.1 – 88.9) | 1.26  (0.99 - 1.60) | 0.061 |
| Withdrawal | 94.5  (89.3 – 99.9) | 88.2  (78.6 – 99.1) | 1.07  (0.95 - 1.20) | 0.233 |
| HTS for TB patients | | | | |
| Lead-in | 93.0  (87.6 – 98.7) | 68.6  (38.1 – 100.0) | 1.36  (0.75 - 2.44) | 0.262 |
| Withdrawal | 96.3  (92.9 – 99.8) | 94.9  (91.1 – 98.8) | 1.01  (0.97 - 1.07) | 0.528 |
| TB screening for PHC clinic attendees | | | | |
| Lead-in | 81.3  (72.9 – 90.6) | 79.2  (66.9 – 93.7) | 1.03  (0.86 - 1.23) | 0.759 |
| Withdrawal | 85.2  (78.7 – 92.2) | 86.6  (80.1 – 93.6) | 0.98  (0.89 - 1.09) | 0.730 |
| ART initiation among HIV-TB co-infected patients | | | | |
| Lead-in | 93.0  (88.2 – 98.0) | 85.5  (70.7 – 100.0) | 1.09  (0.90 - 1.32) | 0.345 |
| Withdrawal | 94.1  (89.7 – 98.6) | 96.5  (93.6 - 99.6) | 0.97  (0.93 - 1.03) | 0.301 |
| IPT among eligible new ART patients | | | | |
| Lead-in | 38.3  (23.8 – 61.6) | 42.1  (27.5 – 64.6) | 0.91  (0.51 - 1.62) | 0.730 |
| Withdrawal | 76.4  (66.3 – 88.1) | 50.8  (36.2 – 71.2) | 1.51  (1.06 - 2.14) | 0.026 |
| VL testing at month 12 after ART initiation | | | | |
| Lead-in | 62.4  (52.7 – 73.9) | 64.6  (58.4 – 71.6) | 0.97  (0.81 - 1.15) | 0.682 |
| Withdrawal | 74.0  (65.5 – 83.6) | 69.9  (63.3 – 77.1) | 1.06  (0.92 - 1.22) | 0.410 |
| Additional outcomes | | | | |
| Confirmed new TB cases % (n/N) | | | | |
| Lead-in | 10.1  (585/5810) | 9.2  (385/4165) | 0.9 | ^✝^ |
| Withdrawal | 7.4  (554/7479) | 6.1  (436/7096) | 1.3 | ^✝^ |
| TB confirmed patients initiated onto TB treatment | | | | |
| Lead-in | 86.0  (503/585) | 90.1  (347/385) | -4.1 | ^✝^ |
| Withdrawal | 85.6  (474/554) | 89.4  (390/436) | -3.8 | ^✝^ |

**Supplementary Table 1: Comparison of HIV-TB service delivery between Quality Improvement and Standard of Care groups in the lead-in and withdrawal phases**

ART, Antiretroviral Therapy; CI, Confidence Interval; HTS, HIV Testing Services; IPT, Isoniazid Preventive Therapy; PHC, Primary Health Care; QI, Quality Improvement; RR, Relative Risk; SOC, Standard of Care; TB, Tuberculosis; VL, viral load

^✝^Only quarterly summary data was available hence no p-value was calculated

**Supplementary Table 2: Resources to scale-up quality improvement to enhance integrated HIV-TB services**

| **Scale-up activity** | **Resource inputs** | **Expected output** | **Expected impact** |
| --- | --- | --- | --- |
| Establish a national leader and co-ordinator for QI scale-up activities, for example, National  TB programme | - Appointment of a QI-trained Project Leader per district to serve as a QI champion and communication liaison for the DoH. - Local NGOs with similar interests in improving HIV-TB integration to support the DoH in the implementation of QI activities and reaching programme targets and milestones. | - SA NDOH-led QI programme which is supported by a network of NGOs. - Common understanding and coordination of QI activities between NGOs and DOH to ensure an effective and productive working relationship. | - Establishment of a sustainable and SA DoH-owned initiative |
| Development of a ‘package’ of best practices for HIV-TB integration | - Input from NGOs, researchers, subject matter experts and implementers on best practices to overcome barriers to integrated HIV-TB service delivery. - An estimated 4 workshops are needed: - Workshop 1: Gather best practices for HIV-TB integration - Workshop 2: Provide feedback on progress and amend or improve best practices. - Workshop 3: An optional periodic workshop to reflect and adapt or expand the best practices package, based on changes in treatment policies, new diagnostics, guidelines, and resources available. - Workshop 4: Finalise best practices package/ share experiences | - A best practices manual for wide-scale rollout of integrated HIV-TB services - Productive partnerships and networks between DOH and NGOs | - A well-informed, sustainable, and supported QI intervention for improving integrated HIV-TB services. |
| Build QI capacity and skills in all relevant stakeholder and implementers | - Identify individuals or organizations with expertise in QI training and implementation. - Adopt a ‘Train the Trainer Model’ to facilitate cascading the training which needs to target: - All levels of leadership in the SA DOH (Upper-, middle- and facility-level leaders) - Key NGO team members - Facility level staff from all categories (i.e., Nurses, Data Capturers, and Lay Counsellors) - Use existing meeting structures (District Management meetings and Performance Feedback meetings) to provide QI training and save on training costs. | - Engaged leadership to implement QI tools and techniques. - A cadre of healthcare worker to implement and cascade QI-training to future healthcare workers - Diversity and representation of all categories of staff in developing QI plans | Skilled healthcare workers to implement QI to enhance integrated HIV-TB services and address weaknesses and gaps in service delivery.  Reduced mortality in HIV-TB patients |
| DQI for routine patient and HIV-TB process data | - Dedicated DQI teams for data quality improvement in every facility, consisting of existing staff from all staff categories. - Assistance from NGOs to address major backlogs in data entry. - Standardize the use of 1-2 unique patient identifiers across all electronic databases for tracking patient results from one database to another. - Direct importing of laboratory results (VL and TB sputum tests) into electronic data bases - Limited or read-only access for all NGOs to summary level data at a facility level, sub-district and district level | - Trustworthy and quality assured data for SA DOH, NGOs and clinic teams to monitor progress. - Patient databases linked by a unique identifier for more accurate analyses and understanding of access to laboratory tests and linkage to treatment for TB and HIV. | Accurate and complete data to identify gaps in services and drive the QI process |
| Wide-spread distribution of user-friendly manuals, training material and tools for facility-level use. | - Development of user-friendly manuals for wide-spread distribution to facilities - Manuals developed by subject matter experts. - Develop online, free access to QI manuals and materials and best practices manuals. | - Manuals developed for:   - QI methods and tools   - Best practices for integrated HIV-TB service delivery - Data Quality Improvement manual | Created awareness of best-practices and demand to be included in QI intervention |

DoH, Department of Health; DQI, Data Quality Improvement; NGO, Non-governmental organization; QI, Quality Improvement; SA, South Africa; TB, tuberculosis; VL, viral load

**Supplementary Figure 1: IPT initiation rates stratified by cluster volume per study group**

CAT=Category; IPT, Isoniazid Preventive Therapy; QI, Quality Improvement; SOC, Standard of Care; I=Intervention, C=Control

**Supplementary Figure 2: HTS performance stratified by cluster volume per study group**

C=Control; CAT= Category; I=Intervention, HTS, HIV Testing Services; QI, Quality Improvement; SOC, Standard of Care;
